# Draft Genome Sequence of Pseudomonas sp. Strain MWU12-2534b, Isolated from a Wild Cranberry Bog in Truro, Massachusetts

**DOI:** 10.1128/MRA.01005-18

**Published:** 2018-10-04

**Authors:** Ghazal Ebadzadsahrai, Jonathon Thomson, Scott Soby

**Affiliations:** a Biomedical Sciences Program, College of Graduate Studies, Midwestern University, Glendale, Arizona, USA; b College of Dental Medicine, Midwestern University, Glendale, Arizona, USA; c Biomedical Sciences Program, College of Graduate Studies and College of Veterinary Medicine, Midwestern University, Glendale, Arizona, USA; Georgia Institute of Technology

## Abstract

An unknown Pseudomonas sp. most closely related to Pseudomonas ficuserectae and Pseudomonas protegens was isolated from the rhizospheres of wild cranberry plants in the Cape Cod National Seashore, in the United States. The draft genome of MWU12-2534b is 6.7 Mbp, has 63.32% GC content, and contains multiple potential virulence and antibiotic resistance genes.

## Abstract (precis)

An unknown Pseudomonas sp. most closely related to Pseudomonas ficuserectae and Pseudomonas protegens was isolated from the rhizospheres of wild cranberry plants in the Cape Cod National Seashore, in the United States.

## ANNOUNCEMENT

**FIG 1:**
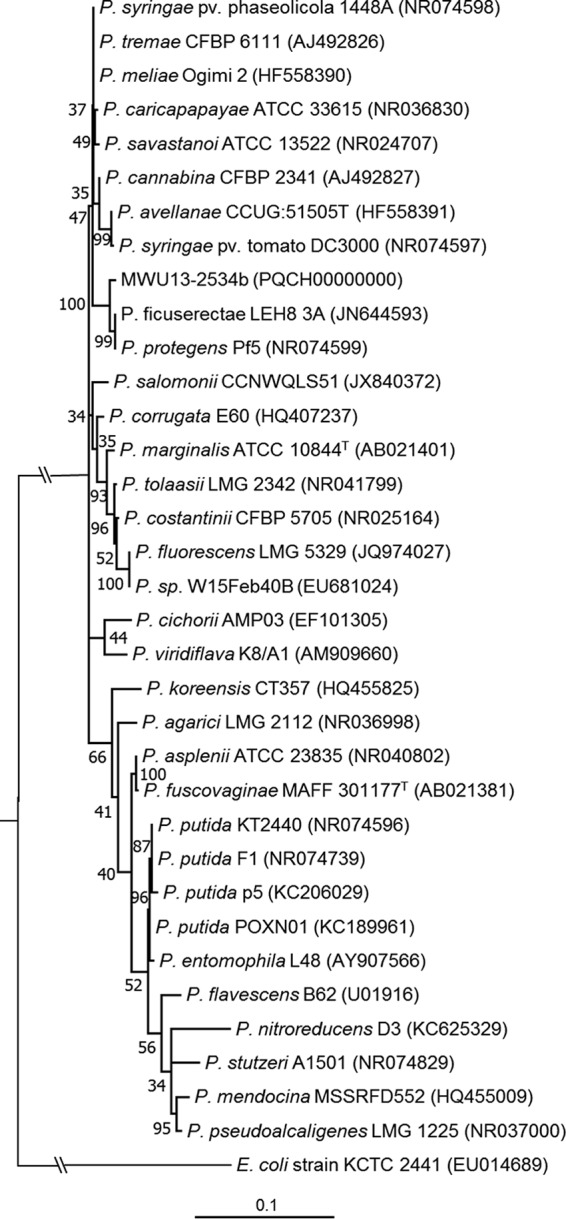
16S rRNA molecular phylogeny of the genus Pseudomonas. The evolutionary history of MWU12-2534b within the genus Pseudomonas was inferred by maximum likelihood using the Kimura 2-parameter substitution model with complete deletion of gaps and missing data. The tree shown is based on a total of 1,319 positions in the final data set, with the highest log likelihood (−4,483.66) shown. The initial tree(s) for the heuristic search was constructed by applying neighbor-joining and BioNJ algorithms to pairwise distances. Gamma distribution was used to model evolutionary rate differences among sites (+G, parameter = 0.1635), allowing for some sites to be evolutionarily invariable ([+I], 58.51% sites). Except for the E. coli outgroup, the tree is drawn to scale, with branch lengths measured in the number of substitutions per site. MWU12-2534b forms a clade with P. ficuserectae and P. protegens but is not a member of either species.

Wetland soil microbes are integral in nutrient cycling and biogeochemical processes, but until recently, little was known about the types of organisms present in these ecosystems. Pseudomonas sp. strain MWU12-2534b was isolated from the rhizospheres of wild cranberry plants in the Cape Cod National Seashore in Massachusetts during a culture-dependent survey of bacteria in bog soils. MWU12-2543b clustered with Pseudomonas ficuserectae and Pseudomonas protegens by 16S rRNA phylogeny (Fig. 1), but genomic comparisons indicate that it belongs within neither of these species. MWU12-2543b had OrthoANI values of 77.32% with P. ficuserectae (NCBI RefSeq accession number NZ_LJQJ01000341) and 89.83% with P. protegens (GenBank accession number MAUL01000003), both under the 94% cutoff for species (1, 2); also, MWU12-2543b had digital DNA-DNA hybridization (dDDH) values of 22.5% with P. ficuserectae and 39.3% with P. protegens, both well below the 70% cutoff for dDDH (3, 4).

Wild cranberry bog soil and root tissue were plated on King’s medium B (KMB) agar containing 50 mg ml^-1^ cycloheximide and ampicillin and grown at 26°C, followed by 3× single-colony purification on KMB agar. Genomic DNA (gDNA) was extracted from overnight KMB broth cultures with a DNeasy blood and tissue kit (Qiagen), and the genome was sequenced at the Arizona State University CLAS Genomics Core facility, first by shearing to ca. 600 bp (Covaris M220 ultrasonicator). Illumina libraries were assembled on an Apollo 384 liquid handler (Wafergen) using a Kapa Biosystems library preparation kit (catalog number KK8201). DNA fragments were end repaired, A tailed, and ligated with combined indexes/adapters (catalog number 520999; Bioo) and then multiplexed into one lane. AMPure beads (catalog number A63883; Agencourt Bioscience/Beckman Coulter, Inc.) were used to clean the adapter-ligated DNA fragments, and then they were amplified with a Kapa HiFi enzyme. An Agilent Bioanalyzer and quantitative PCR (library kit catalog number KK4835; Kapa) were used to assess library quality before pooling and sequencing in 2 × 300- and 2 × 150-bp paired-end flow cells (Illumina MiSeq platform). Read files were then combined, assembled, and annotated on the PATRIC website (http://patricbrc.org) using the Comprehensive Genome Analysis pipeline with default parameters (5, 6). The sequence coverage of 183× allowed partial assembly into 35 contigs totaling 6,738,332 bp, with 63.32% G+C content. The largest contig was 995,949 bp, with an *N*_50_ value of 817,307 bp. The MWU12-2534b genome contained 6,183 coding sequences (CDSs), plus 74 tRNA and 7 rRNA operons. Predicted virulence factor, antibiotic resistance, and efflux pump genes were identified that may contribute to antifungal activity, including genes for proteases, chitinases, hemolysins, hemolytic phospholipases, and nine resistance-nodulation-division (RND) genes (7, 8). GacA (a system that regulates the expression of extracellular enzymes, toxins, quorum-sensing molecules, and motility) (9), ABC molybdenum transporter, a major facilitator superfamily (MFS)-type efflux system, β-lactamases, aminoglycoside *N*(6′)-acetyltransferase, fluoroquinolone resistance, and a type VI secretion system were all found in the genome. Pseudomonas MWU12-2534b has the potential to produce secondary metabolites with antibiotic activity, such as colicin V, pyoverdine, and hydrogen cyanide.

### Data availability.

This whole-genome sequence was deposited at DDBJ/EMBL/GenBank under the accession number PQCH00000000 for Pseudomonas MWU12-2534b. The version described here is version PQCH02000000. The Sequence Read Archive (SRA) is available from GenBank under the accession number SRP154927.
